# The effect of tumor shape irregularity on Gamma Knife treatment plan quality and treatment outcome: an analysis of 234 vestibular schwannomas

**DOI:** 10.1038/s41598-022-25422-9

**Published:** 2022-12-17

**Authors:** Esra Sümer, Ece Tek, O. Artunç Türe, Meriç Şengöz, Alp Dinçer, Alpay Özcan, M. Necmettin Pamir, Koray Özduman, Esin Ozturk-Isik

**Affiliations:** 1 Institute of Biomedical Engineering, Boğaziçi University, Kandilli Campus, Rasathane Cad, 34684 Üsküdar, Istanbul Turkey; 2 Department of Radiation Oncology, School of Medicine, Acıbadem Mehmet Ali Aydınlar University, Istanbul, Turkey; 3 Department of Neurosurgery, School of Medicine, Acıbadem Mehmet Ali Aydınlar University, Istanbul, Turkey; 4 Department of Radiology, Acıbadem Mehmet Ali Aydınlar University, Istanbul, Turkey; 5 Department of Electrical and Electronics Engineering, Boğaziçi University, Istanbul, Turkey

**Keywords:** Surgical oncology, Applied mathematics

## Abstract

The primary aim of Gamma Knife (GK) radiosurgery is to deliver high-dose radiation precisely to a target while conforming to the target shape. In this study, the effects of tumor shape irregularity (TSI) on GK dose-plan quality and treatment outcomes were analyzed in 234 vestibular schwannomas. TSI was quantified using seven different metrics including volumetric index of sphericity (VioS). GK treatment plans were created on a single GK-Perfexion/ICON platform. The plan quality was measured using selectivity index (SI), gradient index (GI), Paddick’s conformity index (PCI), and efficiency index (EI). Correlation and linear regression analyses were conducted between shape irregularity features and dose plan indices. Machine learning was employed to identify the shape feature that predicted dose plan quality most effectively. The treatment outcome analysis including tumor growth control and serviceable hearing preservation at 2 years, were conducted using Cox regression analyses. All TSI features correlated significantly with the dose plan indices (*P* < 0.0012). With increasing tumor volume, vestibular schwannomas became more spherical (*P* < 0.05) and the dose plan indices varied significantly between tumor volume subgroups (*P* < 0.001 and *P* < 0.01). VioS was the most effective predictor of GK indices (*P* < 0.001) and we obtained 89.36% accuracy (79.17% sensitivity and 100% specificity) for predicting PCI. Our results indicated that TSI had significant effects on the plan quality however did not adversely affect treatment outcomes.

## Introduction

Gamma Knife (GK)^1^ is a neurosurgical tool that has been used to treat a wide range of intracranial tumors, including vestibular schwannomas^2,3^, meningiomas^4^, gliomas^5^, and brain metastases^6^, via delivering a precise and high-dose radiation to a target area. Intracranial tumors that are treated using GK radiosurgery exhibit wide variations in volume and shape, and conforming to these variations is a fundamental characteristic of Gamma Knife (GK) dose plans. Over the years, the absolute focus of minimizing radiation exposure to the surrounding brain tissue has led to a dose planning approach in GK that is significantly different from standard radiotherapy planning^7^. An intentional, uneven dose distribution with peak doses within the intracranial target and a steep dose fall-off outside are characteristics of GK dose plans^8,9^. Such conformal plans are achieved with the use of a sphere packing algorithm. Although this algorithm provides great conformality, any irregularity of tumor shape secondarily results in dose heterogeneity within and outside the target. Measuring the effects of tumor shape irregularity (TSI) on dose plan metrics can be useful in clinical decision-making and GK planning, but has not been studied in detail before.

In current GK dose planning practices the primary aim is to conform the radiation dose to the treatment volume (TV) as efficiently as possible. It is widely accepted that the dose delivered to the margin of the TV is the most important variable affecting the clinical outcome and there is wide agreement on the prescription doses to treat each pathology^10^. On the contrary, the guidelines on dose distribution within and outside the target are far less standardized. Conforming the dose to the previously contoured TV is performed manually or supervised by the operator. The planning procedure is guided by several widely accepted treatment quality metrics, which have been extensively studied over the years^11^. Among many others, Selectivity index (SI)^7^, gradient index (GI)^12^, Paddick’s conformity index (PCI)^13^, and efficiency index (EI)^11^ provide useful approximations of how efficiently the radiation dose is concentrated within the TV and how steeply it falls outside the TV (the details of these quality indices are provided in the Supplementary Information). The effect of TSI on the plan quality as measured by the current metrics as well as the dose heterogeneity and the final clinical outcome has remained underexplored. Previous attempts have quantified TSI using radiomics, which transforms medical images into quantitative feature spaces^14–16^. Limkin et al.^17^ reported strong correlations between radiomic shape features and TSI. In addition to the radiomic features, Saad et al.^18^ proposed two- and three-dimensional (2D/3D) shape-based imaging biomarkers and a new metric called ‘volumetric index of sphericity’ (VioS) to represent tumor morphology, and reported VioS as a significant predictor of survival in lung cancer. On the other hand, only a few studies have investigated the effect of shape irregularity and TV on dose plan indices^19,20^. Wu et al. examined the effects of TV and shape complexity on simulated targets, and reported that the ratio of prescription isodose volume to the TV (PITV) and PCI values were higher than the conformity measures for smaller and more irregular targets^19^. More recently, Saraiva et al. suggested that sphericity degree of a target volume can be used to evaluate the dose distribution in GK treatment planning^20^. However, the effects of TV and TSI on GK treatment planning quality and clinical outcome have not been fully investigated.

Vestibular schwannoma is a benign and slow-growing intracranial tumor. When vestibular schwannoma enlarges, the hearing and balance nerves are affected and usually leads to one-sided asymmetric hearing loss and balance^21^. In this study, vestibular schwannomas were used as the tumor model since they have sharp tumor margins on magnetic resonance imaging (MRI) and don’t invade to surrounding tissues.

The aims of this study were threefold: (i) quantifying TSI using radiomic shape features as well as sphericity and VioS, (ii) analyzing the relationships between TSI and core GK dose plan indices (SI, GI, PCI, and EI) and (iii) investigating the impact of TSI on treatment outcomes in vestibular schwannomas.

## Materials and methods

### Subjects

In this retrospective study, 234 vestibular schwannoma patients treated with GK at Acıbadem Mehmet Ali Aydınlar University Altunizade Hospital were included. Thirty-one of these patients underwent previous surgery. The study protocol was approved by the local ethics committee (Acıbadem University and Acıbadem Healthcare Institutions Medical Research Ethics Committee (ATADEK-2020/19)). Written informed consent was obtained from all patients. All methods were performed in accordance with the relevant guidelines and regulations of the approved ATADEK.

### Magnetic resonance imaging

The patients were scanned on a 1.5T or a 3T clinical MR scanner (Siemens Prisma Fit, Erlangen, Germany). The brain tumor MRI protocol included post-contrast (Gadolinium-DTPA) T1-weighted (T1w-Gd) TurboFLASH volumetric imaging (repetition time [TR] = 2080 ms, echo time [TE] = 2.67 ms at 3T; TR = 2240 ms, TE = 3.04 ms at 1.5T) and 3D fluid-constructive interference of steady-state (TR = 5.72 ms, TE = 2.55 ms at 3T; TR = 5.76 ms, TE = 2.58 ms at 1.5T). Each target volume was manually delineated by a neurosurgeon (K.Ö.) with 17 years of experience using Gamma Plan Software (Elekta-Sweden) on T1w-Gd. Three-dimensional models of each tumor were created from the region of interests (ROIs) using 3D Slicer software^22,23^.

### GK treatment and dose plan indices

The GK dose plans were prepared using a Leksell Gamma Plan 10.0 system (Elekta Instrument AB, Stockholm, Sweden) and the treatments were delivered using the Gamma Knife Perfexion or ICON platforms, which are identical in their radiation delivery equipment. GK dose planning is operator dependent, and all planning for this study was performed by a single neurosurgical team. Four dose plan indices including PCI^13^, GI^12^, SI^7^ and EI^11^ were used to evaluate the dose plan quality. Details about calculation of these dose plan indices are provided in the Supplementary Information.

Active cochlear protection was performed in all patients using “dynamic shaping” function of Leksell Gamma Plan 10.0 software and/or margin dose reduction. Maximum cochlear dose was 5.60 [1.50, 16.10] Gy (median and [min,max]).

The possible complications were monitored for the patients with a follow-up period of two years (n = 148). Some complications were observed in a total of nine patients, and included temporary increased gait imbalance (n = 4, 2.70%), dizziness (n = 4, 2.70%), and facial hypoesthesia (n = 3, 2.02%).

### Feature extraction

**Figure 1:**
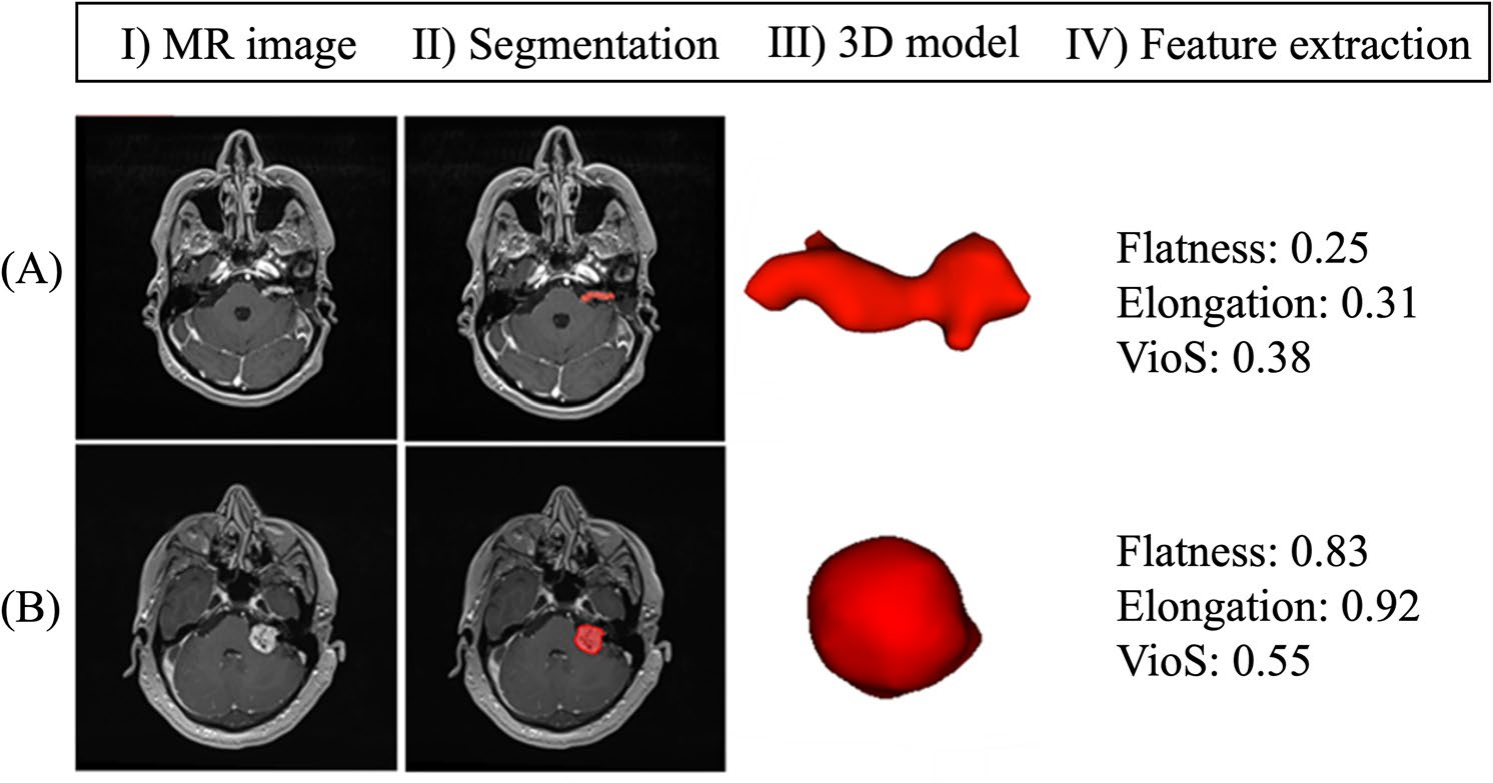
Workflow of the shape feature extraction. (**Ι**) MRI images of two representative vestibular schwannomas. (**II**) The tumor ROIs (red) were defined by an experienced neurosurgeon. (**III**) 3D tumor models constructed using tumor outlining. (**IV**) Three different shape features for tumor A and B. The differences in shape irregularity in the two tumors can be identified by investigating their flatness, elongation, and VioS. Tumor A was flat and irregular, tumor B was more spherical.

SVR, flatness, elongation, spherical disproportion, and compactness were derived to quantify TSI using the Radiomics extension of 3D-Slicer software. Surface to volume ratio (SVR) was normalized (SVR_N_) by multiplying it by the average of the three principal semi-axes of the tumor model (Supplementary Fig. S1), since SVR is proportional to $${\raise0.7ex\hbox{$1$} \!\mathord{\left/ {\vphantom {1 r}}\right.\kern-\nulldelimiterspace} \!\lower0.7ex\hbox{$r$}}$$ Sphericity^20^ and VioS^18^ were computed to show how much a tumor model approaches a sphere. A detailed information about shape irregularity features is provided in the Supplementary Table S1. Figure 1 shows two examples of tumor models and their corresponding shape metrics. Model (A) had an irregular shape (flatness = 0.25, elongation = 0.31, and VioS = 0.38); while model (B) was more spherical (flatness = 0.83, elongation = 0.92, and VioS = 0.55).

### Statistical analysis

The cohort was divided into three TV subgroups (group 1: TV < 1 cm^3^; group 2: 1 < TV < 5 cm^3^; group 3: TV > 5 cm^3^) to evaluate the effect of TV on the shape irregularity features and dose plan indices. A χ^2^ test was performed to compare the sex distributions between subgroups. A Kruskal–Wallis test followed by Dunn’s post-hoc test was used for comparisons of age, dose plan indices, and shape features between subgroups. *P*-values lower than 0.0041 were considered statistically significant for the Kruskal–Wallis test. For Dunn’s test, a *P*-value of < 0.05 was considered statistically significant. All statistical tests were performed using stats model^24^ and scikit-posthocs toolboxes^25^ in Python 3.7.6.

The associations between the TSI and dose plan indices were investigated using a Spearman rank correlation test (r) after multivariate outlier elimination and *P*-values < 0.00125 were considered statistically significant after Bonferroni multiple comparison correction. Feature selection was run for each dose planning index separately using least absolute shrinkage and selection operator (Lasso)^26^. Before Lasso feature selection, multicollinear shape features (r ≥  ± 0.9) were removed. Then, the entire dataset was divided into training (80%) and test (20%) datasets, and the optimal Lasso regularization parameter (λ) was selected by applying threefold cross-validation to the training dataset for each dose planning index. The experiment was run 100 times, and the feature with the overall highest coefficient was selected as the top discriminative feature for each dose planning index. Finally, a univariate linear regression analysis was conducted to define the relationship between the selected feature and the dose planning index of interest. Then, a univariate linear regression analysis was conducted to define the relationship between the selected feature and the dose planning index of interest.

### Machine learning for prediction of dose plan quality

Machine learning algorithms were used to predict high dose plan indices based on the shape features. The expected values of the dose plan indices were set as the median values for the entire patient cohort. Seven shape irregularity features (standardized to zero mean and unit variance) were used as predictor variables, and each dose plan index was used as an independent response variable. Recursive feature elimination (RFE) with a linear kernel support vector machine (SVM) was used to identify the optimal shape features to predict each dose plan index independently^27^. Subsequently, logistic regression (LR), linear discriminant analysis (LDA), gradient boosting (GB), SVM, and random forest (RF) algorithms were used for supervised machine learning. After determining the optimal feature subsets and hyperparameters using training data (80% of the entire data), the model performances were assessed on the independent test set (20% of the entire data). Accuracy, sensitivity, and specificity were calculated for the prediction of each response variable. All machine learning implementations were run in Python using the Scikit-learn library.

### Treatment outcome analysis

“Tumor growth control” (no persistent volumetric tumor growth at 2 years after treatment; data available for 148 primary vestibular schwannomas out of 234 patients) and “serviceable hearing preservation” (defined as maintenance of Gardner-Robertson class I or II hearing function at 2 years; data available for 83 patients with primary tumors) were used as outcome measures. The sex distribution differences between patients with persistent tumor growth or no growth, and patients with serviceable hearing preservation or loss were compared using a χ^2^ test. Univariable and multivariable Cox proportional hazards models were also used to evaluate the influence of TSI on tumor control and serviceable hearing preservation. For tumor control, the model included following variables: age, sex, TV, Koos grade, margin dose, coverage index (CI), SI, GI, EI and VioS. For serviceable hearing preservation, maximum cochlear dose, cochlea-target distance and radiation dose to tumor margin were added to the above variables. Additionally, the respective variables were compared between the subgroups of tumor growth and serviceable hearing using a Mann–Whitney U test.

### Conference presentation

The partial results of this study were presented as an electronic poster at the Society of Neuro-Oncology (SNO) 25^th^ Annual Meeting 2020.

## Results

### Patient population, dose plan indices, and shape irregularity features

Table 1 shows the demographics and the median [range] dose planning indices of the vestibular schwannoma cohort of this study. The TV values ranged from 0.01 to 20.01 cm^3^, which were divided into low (group 1, TV < 1 cm^3^, 83 patients), medium (group 2, 1–5 cm^3^, 89 patients), and high (group 3, > 5 cm^3^, 62 patients) TV groups. Sex distribution and age were similar in all subgroups (*P* = 0.897 and *P* = 0.767, respectively). Group 1 had statistically significantly lower SI (*P* < 0.001), lower PCI (*P* < 0.00) and EI (*P* < 0.001), and higher GI (*P* < 0.001) than groups 2 and 3. Similarly, group 2 patients had lower SI (*P* = 0.0003), PCI (*P* = 0.0005), and EI (*P* = 0.0024), and higher GI (*P* = 0.007) than group 3. Therefore, SI, PCI, and EI were higher, and GI was lower in large TVs.

**Table1:** Patient characteristics and summary of dose planning indices of this study.

	TV < 1 cm^3^ (Group 1)	1 cm^3^ < TV < 5 cm^3^ (Group 2)	TV > 5 cm^3^ (Group 3)	*P*-value	Overall
Number of patients (M, F)	83 (38, 45)	89 (39,50)	62 (26,36)	*P* = 0.897	234 (103,131)
Age Median [Range]	52 [21,78]	50 [18,87]	47 [18,89]	*P* = 0.767	50 [18,89]
SI Median [Range]	0.74 [0.2, 0.96]	0.86[0.57,0.96]	0.91[0.75,0.99]	*P* = 2.4E-19, 1 vs 2**, 1 vs 3**, 2 vs 3**	0.80 [0.20, 0.99]
PCI Median [Range]	0.70 [0.20, 083]	0.83 [0.57,0.96]	0.89 [0.62,0.94]	*P* = 1.5E-24 1 vs 2**, 1 vs 3**, 2 vs 3**	0.80 [0.20,0.96]
GI Median [Range]	2.83 [2.5, 4.00]	2.68 [2.40, 3.39]	2.54 [2.41,2.99]	*P* = 8.4E-17 1 vs 2**, 1 vs 3**, 2 vs 3*	2.70 [2.40, 4.00]
EI Median [Range]	0.41 [0.12, 0.63]	0.49 [0.30, 0.58]	0.52 [0.02, 0.59]	*P* = 1.5E-18 1 vs 2**, 1 vs 3**, 2 vs 3*	0.48 [0.02,0.63]

**Figure 2:**
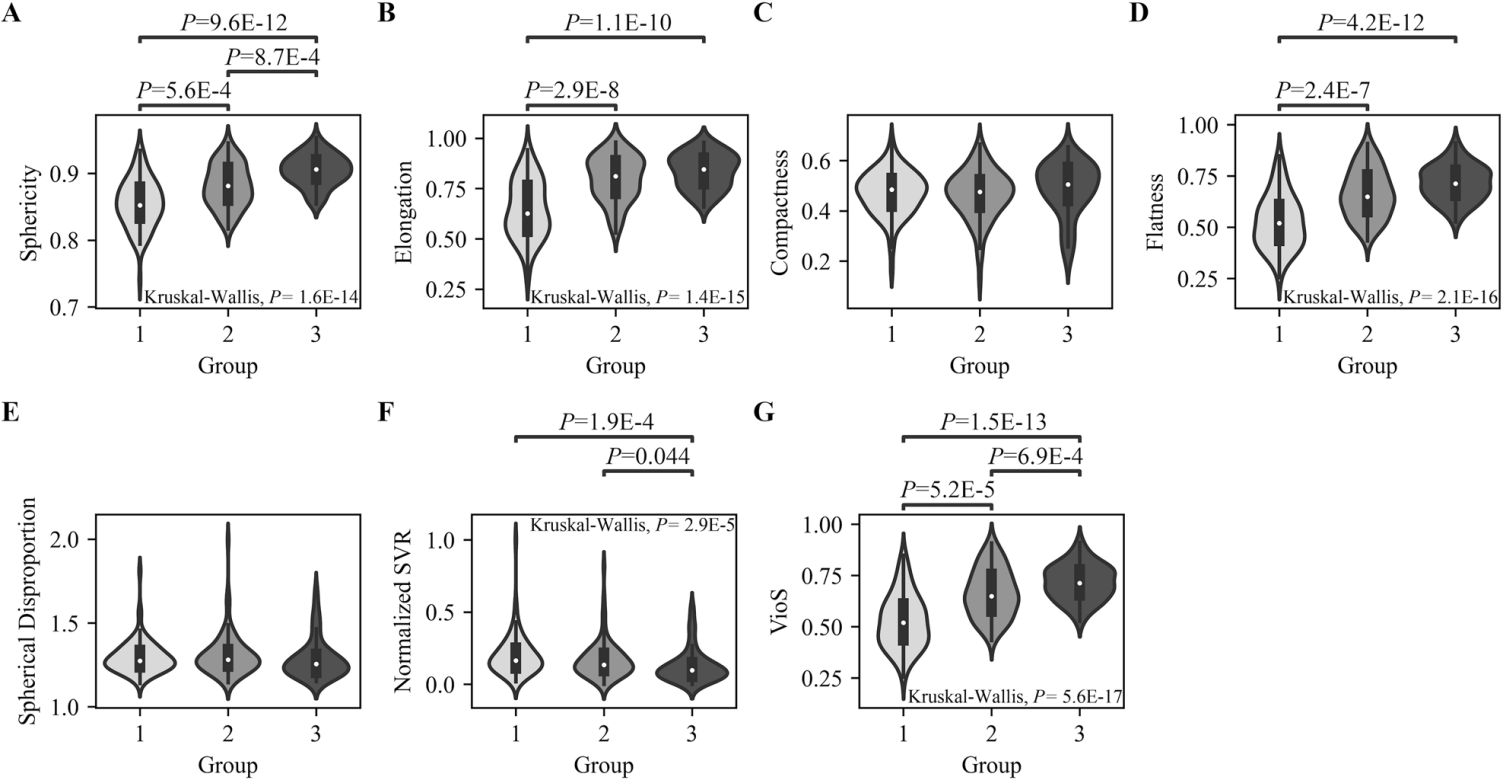
The violin plots outline the median value (white dot) and interquartile range (thick gray bar) of the shape irregularity features: (**A**) sphericity, (**B**) elongation, (**C**) compactness, (**D**) flatness, (**E**) spherical disproportion, (**F**) SVR_N_, and (**G**) VioS for different TV subgroups. The *P*-values were Bonferroni adjusted and obtained using a Kruskal–Wallis test followed by Dunn’s post-hoc test.

Figure 2 shows the distributions of the shape features in the TV subgroups. Compactness (*P* = 0.106) and spherical disproportion (*P* = 0.105) were not significantly different between subgroups. Group 3 had the highest sphericity, elongation, flatness, and VioS, and the lowest SVR_N__._ Additionally, Group 1 had lower sphericity, elongation, flatness, and VioS than groups 2 and 3 (*P* < 0.001 for all) and higher SVR_N_ than group 3 (*P* < 0.001). In contrast, group 2 had significantly higher sphericity, elongation, flatness, and VioS than group 1 (*P* < 0.001 for all) and lower sphericity and VioS than group 3 (*P* < 0.001 for both).

### Correlations between dose plan indices and shape irregularity features

The Spearman correlation coefficients (*r*) of the association between the TSI metrics and GK planning indices are provided in Table 2. In the overall patient cohort, all shape features had statistically significant correlations with the dose plan indices (*P* < 0.001). SVR had the highest correlation with all the plan indices; the corresponding correlation values were *r* =  − 0.74, *r* = 0.62, *r* =  − 0.72, and *r* =  − 0.79 for SI, GI, EI, and PCI, respectively (*P* < 0.00125 for all). Although the correlations decreased after normalization, SVR_N_ still showed significant correlations with SI, GI, EI, and PCI (*r* =  − 0.53, *r* = 0.4, *r* =  − 0.49, *r* =  − 0.59, respectively; *P* < 0.001). Flatness had a direct relationship with SI, EI and PCI (*r* = 0.55, *r* = 0.53, *r* = 0.61, respectively; *P* < 0.001) and an inverse relationship with GI (*r* =  − 0.48, *P* < 0.001). The results showed that elongation had a direct relationship with SI (*r* = 0.46, *P* < 0.001), EI (*r* = 0.49, *P* < 0.001), and PCI (*r* = 0.53, *P* < 0.001), and an inverse relationship with GI (*r* =  − 0.37, *P* < 0.001). Spherical disproportion was consistently associated with SI (*r* =  − 0.44), GI (*r* = 0.23), EI (*r* =  − 0.39), and PCI (*r* =  − 0.46; *P* < 0.001 for all). Similarly, compactness was significantly related to the dose plan indices (*r* = 0.42, *r* =  − 0.27, *r* = 0.43, and *r* = 0.5 for SI, GI, EI, and PCI, respectively; *P* < 0.001). GI and sphericity were negatively correlated (*r* =  − 0.52, *P* < 0.001), whereas SI, EI, and PCI were positively correlated (*r* = 0.55, *r* = 0.57, *r* = 0.63, respectively; *P* < 0.001). A scale-independent shape feature, VioS, had correlations higher than 0.6 with SI, EI, and PCI (*r* = 0.63, *r* = 0.69, *r* = 0.68, respectively; *P* < 0.001 for all), and a negative correlation with GI (*r* =  − 0.58, *P* < 0.001). The last column of Table 2 shows the correlations between shape features and TV. TV was strongly correlated with SVR (*r* =  − 0.98, *P* < 0.001) and moderately to SVR_N_ (*r* =  − 0.36). TV was significantly related to elongation, flatness, sphericity, and VioS (*r* = 0.52, *r* = 0.59, *r* = 0.53, and *r* = 0.6) but not to spherical disproportion and compactness (*r* =  − 0.13, *P* = 0.06 and *r* = 0.17, *P* = 0.02, respectively). The results suggested that SI, EI, and PCI increased while GI decreased as the tumor shape became more spherical. Additionally, larger vestibular schwannomas presented more regular shapes.

**Table 2:** Spearman correlation analysis of all tumor models (overall) and subgroups (Group 1/2/3) for evaluating the relationship of each shape feature with dose planning indices (*P < 0.00125).

Shape features		Treatment Parameters				
		Spearman correlation coefficient *(r)*				
		SI	GI	EI	PCI	TV
Surface to volume ratio (SVR)	Overall	− 0.74*	0.62*	− 0.72*	− 0.79*	− 0.98*
	Group 1/2/3	− 0.44*/ − 0.5*/ − 0.41	0.55*/0.33/0.23	− 0.64*/ − 0.57*/ − 0.42	− 0.54*/ − 0.54*/ − 0.53*	− 0.94*/ − 0.92*/ − 0.72*
Normalized surface to volume ratio (SVR_N_)	Overall	− 0.53*	0.4*	− 0.49*	− 0.59*	− 0.36*
	Group 1/2/3	− 0.28/ − 0.47*/ − 0.41	0.16/0.2/0.39	− 0.27/ − 0.53*/ − 0.62*	− 0.33/ − 0.48*/ − 0.59*	0.01/ − 0.27/0.09
Flatness	Overall	0.55*	− 0.48*	0.53*	0.61*	0.59*
	Group 1/2/3	0.21/0.34/0.27	− 0.38*/ − 0.11/ − 0.41	0.33/0.42*/0.44	0.31/0.38*/0.41	0.25/0.3/ − 0.09
Elongation	Overall	0.46*	− 0.37*	0.49*	0.53*	0.52*
	Group 1/2/3	0.15/0.23/0.06	− 0.32/ − 0.15/ − 0.24	0.17/0.36*/0.2	0.25/0.32/0.2	0.15/0.27/0.16
Spherical disproportion	Overall	− 0.44*	0.23*	− 0.39*	− 0.46*	− 0.13
	Group 1/2/3	− 0.24/ − 0.47*/ − 0.42	0.05/0.16/0.4	− 0.22/ − 0.47*/ − 0.65*	− 0.28/ − 0.57*/ − 0.62*	0.26/ − 0.19/0.14
Compactness	Overall	0.42*	− 0.27*	0.43*	0.5*	0.17
	Group 1/2/3	0.21/0.49*/0.46	− 0.03/ − 0.16/ − 0.4	0.22/0.49*/0.72*	0.28/0.57*/0.62*	− 0.24/0.24/ − 0.16
Sphericity	Overall	0.55*	− 0.52*	0.57*	0.63*	0.53*
	Group 1/2/3	0.23/0.28/0.42	− 0.24/ − 0.36*/ − 0.43	0.3/0.46*/0.54*	0.3/0.37*/0.52*	− 0.03/0.41*/0.19
Volumetric index of sphericity (VioS)	Overall	0.63*	− 0.58*	0.69*	0.68*	0.6*
	Group 1/2/3	0.25/0.37*/ 0.38	− 0.26/ − 0.34/ − 0.41	0.27/0.54*/0.55*	0.33/0.38*/0.55*	0.095/0.35*/0.07

### Correlations between dose plan indices and shape irregularity features in patient subgroups stratified by tumor volume

The results of the correlation analyses in the TV subgroups are provided in Table 2. Group 1 showed significant correlations only between SVR and the plan indices (*r* =  − 0.44, *r* = 0.55, *r* =  − 0.64, *r* =  − 0.54 for SI, GI, EI, and PCI, respectively; *P* < 0.001). In group 2, SVR_N_ consistently had significant associations with the dose plan indices (*r* =  − 0.47, *r* =  − 0.53, *r* =  − 0.48 for SI, EI, and PCI, respectively; *P* < 0.001), except GI (*r* = 0.2, *P* = 0.07). Flatness was negatively associated with GI (*r* =  − 0.38, *P* = 0.0006) in group 1 and positively correlated with EI (*r* = 0.42, *P* < 0.001) and PCI (*r* = 0.38, *P* = 0.0004) in group 2. Only EI was significantly associated with elongation in group 2 (*r* = 0.36, *P* = 0.001). EI and PCI were negatively correlated with spherical disproportion in groups 2 and 3 with lower than − 0.47 correlation coefficients (*P* < 0.001 for both). Compactness, sphericity, and VioS had positive correlations with EI and PCI in groups 2 and 3, with correlation coefficients ranging from 0.37 to 0.72 (*P* < 0.001). Despite the significant correlations between the shape irregularity features and TV in the overall patient cohort, TV was not significantly correlated to the shape irregularity features in most subgroups. Only SVR had consistent correlations with TV in the subgroups. Additionally, the increments in sphericity and VioS were correlated with TV increments only in group 2 (*r* = 0.41, *P* = 0.0001, and *r* = 0.35, *P* = 0.001, respectively). Meanwhile, the results suggested that the correlation coefficients between the shape irregularity features, excluding SVR, and the dose plan indices increased as the tumors got larger. SVR_N_ and compactness had stronger correlations with GI, EI, and PCI. Additionally, flatness and spherical disproportion showed stronger correlations with dose plan indices in higher volume subgroups. The correlation coefficients of sphericity and VioS shape features with all the dose plan indices also increased consistently from group 1 to group 3.

### Associations of shape irregularity features and dose planning indices

**Figure 3:**
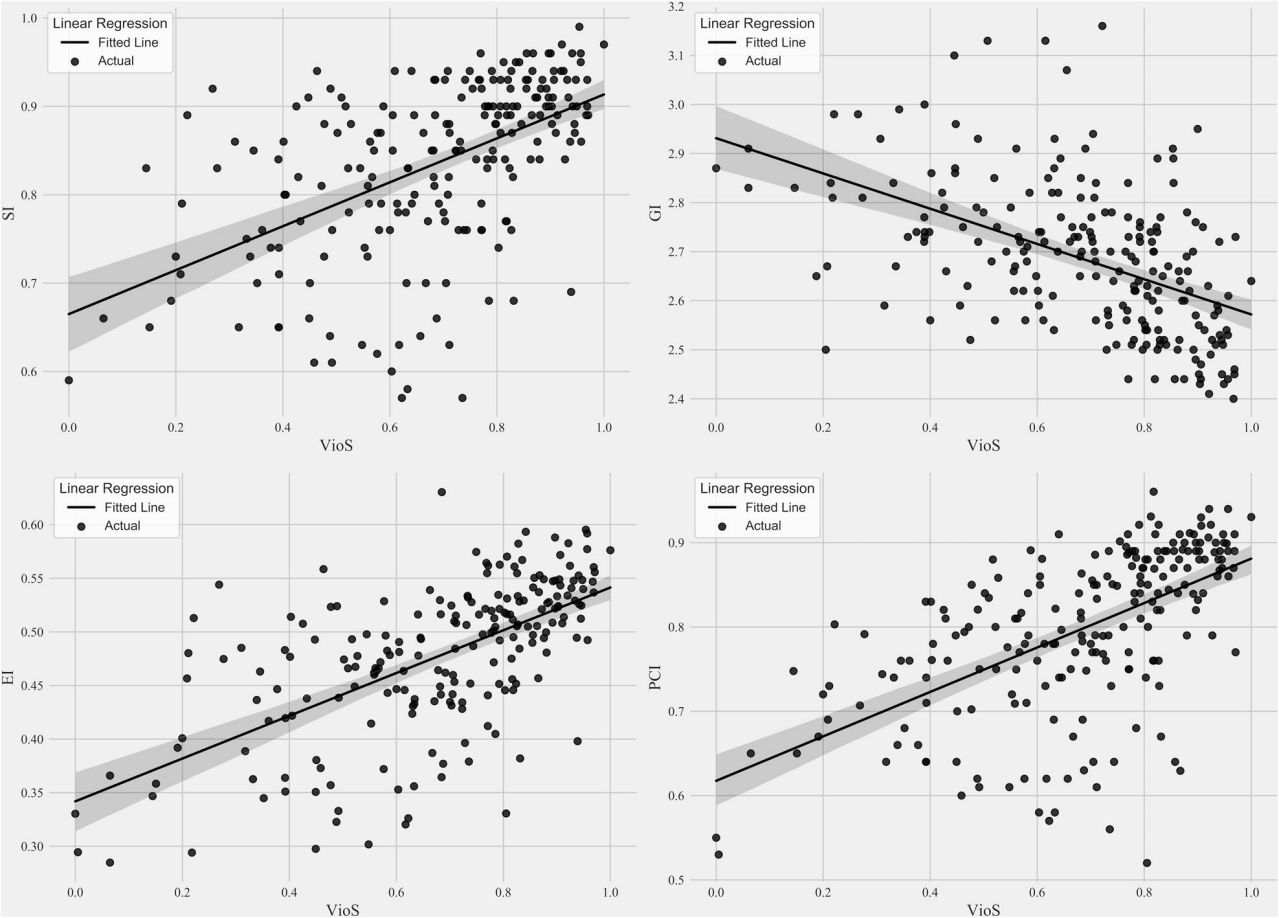
The univariate linear regression analysis results for the correlation of VioS with SI, GI, EI, and PCI.

Lasso feature selection identified VioS as the most associated feature with all the dose plan indices. Supplementary Table S2 shows the univariate multiple linear regression analysis results for predicting dose indices based on VioS. VioS had statistically significant linear relationships with SI, GI, EI, and PCI with adjusted *R*^*2*^ values of 0.27, 0.24, 0.38, and 0.33, respectively (*P* < 0.001 for all models). The scatter plots of VioS and dose plan indices with fitted regression lines and 95% confidence intervals are shown in Fig. 3.

### Feature selection and machine learning models

Table 3 shows the best classification results for each dose plan index using either VioS or the features selected by combination of recursive feature elimination and support vector machine (RFE-SVM). While RFE-SVM selected only VioS as the best predictive feature for SI, the effective features for predicting GI were VioS, elongation, flatness, compactness, and sphericity. Similarly, VioS and compactness were predictive features for EI. On the other hand, the predictive feature subset of PCI consisted of VioS, sphericity, flatness, compactness, and SVR_N_. The highest training accuracy for predicting EI and PCI was 90.37% using a RF. The highest classification accuracy of 74.47% (sensitivity = 64%, specificity = 86.36%) was obtained for predicting SI using the selected features of VioS with GB on the test dataset. On the other hand, an accuracy of 74.47% (sensitivity = 72%, specificity = 77.27%) was obtained for predicting GI. The highest classification accuracy for predicting EI was 82.98% (sensitivity = 84%, specificity = 81.82%). The highest accuracy of 85.11% was achieved with an RF classifier (sensitivity = 79.17%, specificity = 91.30%) for predicting PCI.

**Table 3:** Best classification results for each dose plan indices using either the features selected by RFE-SVM or VioS in the training and test datasets.

	Selected Features	Class. Method	n	Acc/Sens/Spec (%)
SI	VioS	GB	Training n_0_ = 90, n_1_ = 97	73.8/73.20/74.44
			Testing n_0_ = 22, n_1_ = 25	74.47/64/86.36
GI	VioS, elongation, flatness, compactness, sphericity	RF	Training n_0_ = 88, n_1_ = 99	80.21/ 80.81/ 79.55
			Testing n_0_ = 22, n_1_ = 25	74.47/ 72/ 77.27
EI	VioS, compactness	RF	Training n_0_ = 90, n_1_ = 97	90.37/ 88.66/ 92.22
			Testing n_0_ = 23, n_1_ = 24	82.98/ 84/ 81.82
PCI	VioS, sphericity, flatness, compactness, SVR_N_	RF	Training n_0_ = 90, n_1_ = 97	90.37/ 91.75/ 88.89
			Testing n_0_ = 23, n_1_ = 24	85.11/ 79.17/ 91.30
SI	VioS	GB	Training n_0_ = 90, n_1_ = 97	73.8/73.20/74.44
			Testing n_0_ = 22, n_1_ = 25	74.47/64/86.36
GI	VioS	RF	Training n_0_ = 88, n_1_ = 99	78.61/77.78/79.55
			Testing n_0_ = 22, n_1_ = 25	72.34/68/77.27
EI	VioS	GB	Training n_0_ = 90, n_1_ = 97	77.54/73.20/82.22
			Testing n_0_ = 23, n_1_ = 24	80.85/76/86.36
PCI	VioS	GB	Training n_0_ = 90, n_1_ = 97	74.33/68.04/81.11
			Testing n_0_ = 23, n_1_ = 24	89.36/79.17/100

VioS was selected as a predictive feature for all dose plan indices. Therefore, the machine learning models were further trained with only VioS, and their performances were evaluated on the test set. With VioS, we observed slightly decreased accuracies for the predictions of GI and EI. In contrast, the accuracy for predicting PCI increased to 89.36% (sensitivity = 79.17%, specificity = 100%) using VioS as the only feature with GB.

### The effects of VioS and dose plan indices on treatment outcome

**Figure 4:**
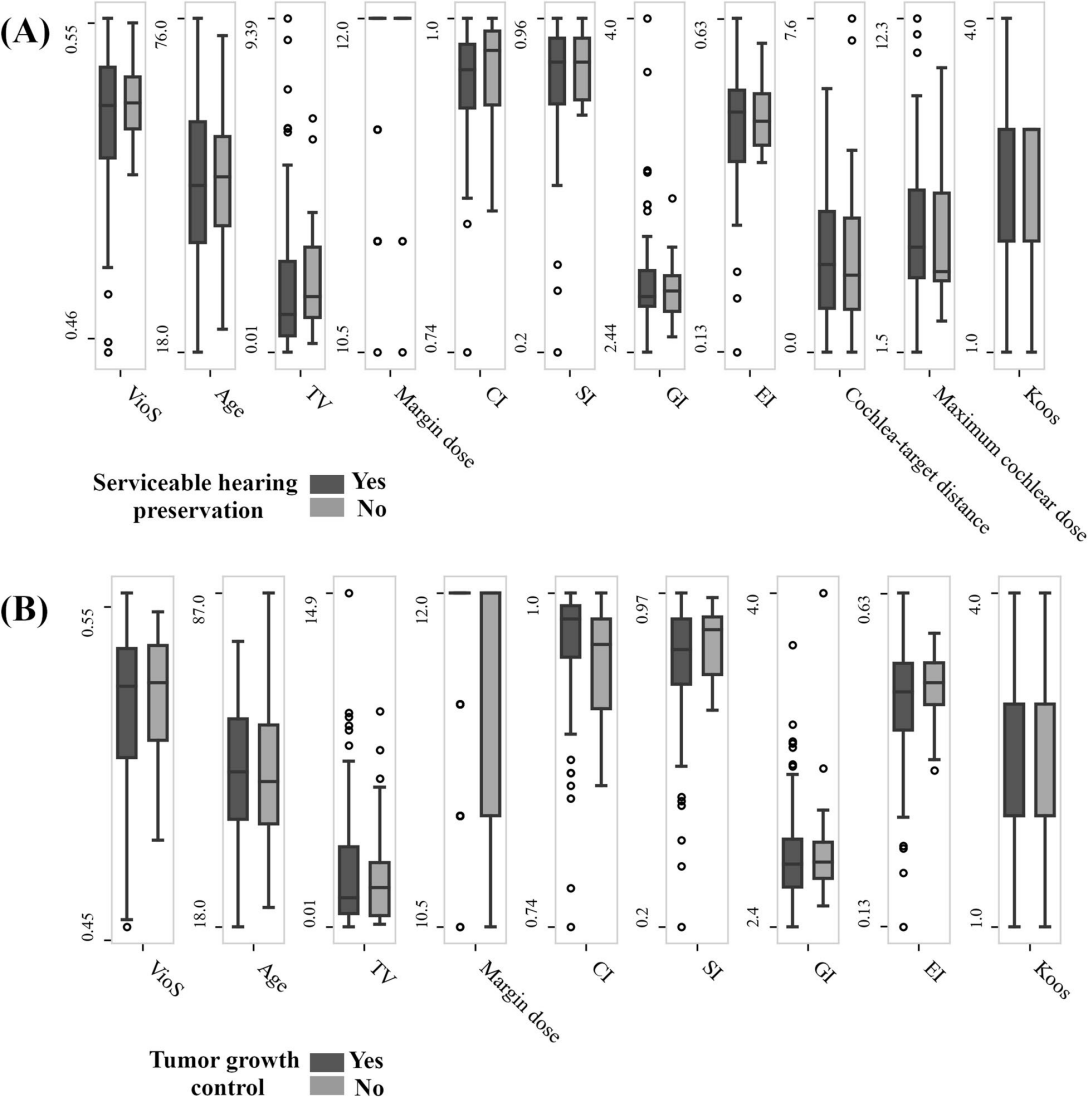
Comparisons of clinical and treatment parameters of vestibular schwannoma patients with or without hearing preservation (**A**) and tumor growth control (**B**).

Table 4 shows the summary of clinical and treatment parameters of the vestibular schwannoma patients with 2-years follow-up. Sex and age distributions did not significantly differ between patients with serviceable hearing preservation or loss (*P* = 0.64 and *P* = 0.38 respectively). Clinical and treatment variables also were similar as shown in Fig. 4A. Following GK treatment, serviceable hearing preservation rate at 2-years was approximately 75.9%. Univariable and multivariable Cox regression analyses showed that none of the covariates had an effect on hearing preservation (*P* > 0.05 for all) as presented in Supplementary Table S3 Similarly, sex and age distributions didn’t significantly differ between patients with or without tumor growth control (*P* = 0.41 and *P* = 0.38 respectively). Additionally, Fig. 4B shows clinical and treatment parameters of patients with or without tumor growth control were similar. At two years after GK treatment, the tumor growth control rate was about 85.1%. Multivariable Cox analysis confirmed that margin dose was associated with increased tumor growth control at two years (Supplementary Table S4).

**Table 4:** The demographics information, clinical assessment parameters and dose plan indices of the patients included in the outcome analysis.

Patient characteristics	
Number of patients (M, F)	148 (69, 79)
Age Median [Range]	50 [87, 18]
TV (cm^3^) Median [Range]	1.41 [0.015, 14.86]
**Koos grade value (%)**	
I	21(14.2%)
II	59 (39.9%)
III	49 (33.1%)
IV	19 (12.8%)
**Gardner-Robertson Class**	
I	31 (21%)
II	52 (35.1%)
III, IV, and V	65 (43.9%)
**GK Treatment Parameters**	**Median [Range]**
SI	0.85 [0.20, 0.97]
CI	0.97 [0.74, 1.00]
GI	2.70 [2.40, 4.00]
EI	0.48 [0.13, 0.63]
PCI	0.79 [0.20, 0.96]
Central dose (Gy)	24.00 [20.00–30.00]
Margin dose (Gy)	12.00 [10.50, 12.00]
Cochlea- target distance (mm)	1.80 [0.00,10.00]
Maximum cochlear dose (Gy)	5.60 [1.50, 16.10]
Observation period (months)	24 [24, 52]

## Discussion

GK radiosurgery is an established technology for delivering radiation selectively to intracranial targets. The primary aim of GK dose planning is to conform to any irregularities of the target shape. GK dose planning protocols are best suited for spherical targets, but most intracranial pathologies have irregular shapes^28^. Additionally, GK dose planning becomes more complex with increasing irregularity, resulting in a heterogeneous dose distribution within the target and in surrounding areas^28–31^. In this study, we analyzed the effects of tumor shape irregularity on dose plan quality and identified significant parameters affecting treatment outcome in vestibular schwannomas. We used seven different metrics to quantify tumor shape irregularity ranging from basic methods such as surface to volume ratio, flatness, elongation, spherical disproportion, compactness, and sphericity to newer methods such as VioS. Our results indicated that the radiomics-based shape irregularity features and treatment plan quality were significantly correlated. Additionally, irregularly shaped and smaller tumors had lower SI, EI, PCI, and higher GI, indicating worse plan quality. Although the TSI didn’t have a significant effect on treatment outcomes, the margin dose was defined as a significant factor affecting tumor growth control at two years. Moreover, machine learning algorithms succesfully predicted higher dose plan indices based on tumor shape features.

Although it is universally accepted that the dose distribution is affected by the shape and size of the target, few authors have attempted to define the effect of TSI on radiosurgery dose plan indices^19,20^. Wu et al.^19^ created mathematical simulations of targets with different degrees of irregularity and concluded that the TSI was inversely correlated with PCI. Additionally, Chagas-Saraiva et al.^20^ reported that tumor sphericity degree facilitated the assessment of the dose distribution within the target. Similarly, our results indicated that irregular tumors had less conformal dose distrubution.

Radiomics is a relatively new field that has been scarcely applied to vestibular schwannomas. Previous studies have mainly focused on predicting the vestibular schwannoma growth rate^32^, transient enlargement^33^, prognosis^34^, and treatment response^35^ using radiomics features. According to our results, the TSI features were not only correlated with dose plan indices, but also had the potential to predict the treatment plan quality in vestibular schwannomas. Especially, VioS was identified as a strong predictor of plan quality using machine learning algorithms. Predicting the highest possible dose plan quality for a given tumor shape may be relevant in the clinical practice as GK treatment planning is still a manual process.

Previous studies on different pathologies have reported conflicting results on the effect of TV on the dose plan indices^36,37^. Menon et al.^37^ reported that PCI was not influenced by the target volume changes in 60 cerebral arteriovenous malformations. In contrast, Stanley et al.^36^ indicated that PCI was not independent of TV. Additionally, we observed that the dose plan indices were highly dependent on TV, and while SI, EI, and PCI were higher, GI was lower in larger tumors. These discrepancies may be explained by the lesion differences and diverging planning strategies^38^. In contrast to malignant tumors or arteriovenous malformations, vestibular schwannomas are benign tumors that grow primarily by expansion, without invading the surrounding tissues, explaining their more spherical shape with increasing TV. This was an important confounding factor in our study, because a more spherical tumor shape resulted in improved plan quality metrics for larger tumors. The SVR estimation not only depended on but had strong correlations with TV. To overcome this limitation, we normalized TV-dependent shape features before the analysis. Nevertheless, the shape features, except the compactness and spherical disproportion, were still significantly correlated with TV, indicating that larger vestibular schwannomas had a more spherical or less irregular shape.

There is great variability in size of vestibular schwannomas at the time of GK treatment and the dose plan indices are affected significantly by the treatment volume. At least two different effects are observed: First of all, treatment quality metrics perform less than optimal in very small tumors^36,39^. Similarly, Lomax et al.^38^ reported that the conformity index decreased with TV in tumors smaller than 1 cm^3^. Additionally, tumors with TV < 0.2 cm^3^ were excluded from another study^12^. Moreover, Wu et al.^19^ analyzed the effects of TV on two conformity measures, PITV and PCI, and concluded that conformity is reduced in smaller targets. Secondly, dose plan indices improve in larger tumors despite a higher dose to the surrounding tissues^12,40^. This results in higher complication rates despite better quality metrics. To overcome these paradoxical effects, we systematically analyzed the relationships between TV and dose plan indices by further subdividing our cohort into small-, medium- and large-volume subgroups. In line with the previous studies’ findings, we noticed that tumors with smaller TVs had worse plan quality indices than larger tumors.

Tumor growth control was chosen to assess the efficiency of the treatment and serviceable hearing preservation was chosen as a measure of safety of the treatment. GK treatment rates were comparable to the values reported in the current literature^41^. We also tested the effect of TSI on the treatment outcome. A previous large cohort study reported that serviceable hearing preservation, clinical tumor control, and radiographic tumor control were not affected by any factors including patient age and sex, laterality, tumor grade, prescription isodose, mean, maximum, and minimum target doses, RTOG conformity and homogeneity indices, energy index, SI, and PCI^42^. Additionally, they reported that lower GK dose rates (< 2.67 Gy/min) were associated with better freedom from progressive symptomatic hearing loss and functional nerve dysfunction. In contrast, Brown et al. reported that coverage and age had adverse effects on hearing loss^43^. According to our results, TSI did not have a significant impact on tumor growth control or serviceable hearing preservation at two years after GK. It seems that low TSI of a benign and non-infiltrative tumor would not significantly impact the ability of GK technology. Previously, it was shown that GK significantly reduced the dose to critical structures by using its dynamic shaping feature while delivering treatment^44^. Recently, Paddick et al. compared GK with different SRS platforms and revealed that the extracranial dose was the lowest for GK^45^. Thus, GK delivers the necessary dose to the whole tumor while limiting the amount of radiation delivered to the surrounding inner ear structures. Another reason might be that the operator could manually compensate for the dose heterogeneity caused by TSI by manipulating the treatment plan. Even if a minor effect on treatment outcome arised from TSI, one should keep in mind that our sample size might be limited to show such an effect. On the other hand, we identified higher margin dose as a significant factor affecting tumor growth control which had already been proven in other studies^10^.

This study had also some limitations. There is no ideal machine learning method that provides the best solution to every problem^46^. For this study, we investigated five different state-of-the-art classifiers with a feature selection process, RFE-SVM^47^, which is one of the most popular algorithms for selecting the best feature subset. However, we did not observe higher accuracies for all the response variables after feature elimination due to the limited number of shape irregularity features. Other feature selection and machine learning algorithms may be tested on a larger feature set for improved prediction accuracy. Moreover, the quantification of shape irregularity is a fundamental problem in mathematics, and there is no single perfect measure. Therefore, we chose a comprehensive approach and analyzed seven different shape metrics in a large and homogenous patient cohort. Furthermore, although the treatment quality indices used in this study are widely accepted, they are mere mathematical models and do not necessarily reflect the biological reality.

## Conclusion

In conclusion, TSI was identified as a significant factor affecting GK dose plan quality but not the treatment outcomes in vestibular schwannomas. With increasing size, tumors become more spherical resulting in higher plan quality. Among several shape irregularity metrics, VioS was the strongest predictor of GK plan quality in vestibular schwannomas. Consequently, quantifying tumor shape irregularity and predicting dose plan indices beforehand could provide the clinician with an initial estimation of achievable plan quality during manual dose planning. Further analyses using different pathologies, different operators, and prospective study designs may facilitate a better understanding of the effect of TSI on GK dose planning.

## Supplementary Information

Supplementary Information.

## Data Availability

The datasets generated during and/or analyzed during the current study are available from the corresponding author on reasonable request.
