# Supplementary material for: The effect of tumor shape irregularity on Gamma Knife treatment plan quality and treatment outcome: an analysis of 234 vestibular schwannomas
Source: Sci Rep. 2022 Dec 17;12:21809. doi: 10.1038/s41598-022-25422-9 (PMC9759589; doi:10.1038/s41598-022-25422-9)
Supplement: Supplementary file 1 — Supplementary Information. [file 41598_2022_25422_MOESM1_ESM.pdf]

Title: The Effect of Tumor Shape Irregularity on Gamma Knife Treatment Plan Quality and Treatment Outcome: An Analysis of 234 Vestibular Schwannomas

Complete Author List

1. Esra Sümer, MSc, Institute of Biomedical Engineering, Bogazici University, Istanbul, Turkey
2. Ece Tek, Med. Phys., Department of Radiation Oncology, Acıbadem Mehmet Ali Aydınlar University, School of Medicine, Istanbul, Turkey
3. O. Artunç Türe, Med.Phys., Department of Radiation Oncology, Acıbadem Mehmet Ali Aydınlar University, School of Medicine, Istanbul, Turkey
4. Meriç Şengöz, MD, Department of Neurosurgery, Acıbadem Mehmet Ali Aydınlar University, School of Medicine, Istanbul, Turkey
5. Alp Dinçer, MD, Department of Radiology, Acıbadem Mehmet Ali Aydınlar University, School of Medicine, Turkey
6. Alpay Özcan, PhD, Department of Electrical and Electronics Engineering, Boğaziçi University, Istanbul, Turkey
7. M. Necmettin Pamir, MD, Department of Neurosurgery, Acıbadem Mehmet Ali Aydınlar University, School of Medicine, Istanbul, Turkey
8. Koray Özduman, MD, Department of Neurosurgery, Acıbadem Mehmet Ali Aydınlar University, School of Medicine, Istanbul, Turkey
9. Esin Ozturk-Isik, PhD, Institute of Biomedical Engineering, Bogazici University, Istanbul, Turkey

Dose Plan Indices

Paddick conformity index (PCI) is a conformity measure that considers the irradiated proportion of normal tissues [1], estimated using the following formula:

$$\mathrm{PCI} = \frac{\mathrm{TV}_{\mathrm{PIV}}^2}{\mathrm{TV} \times \mathrm{PIV}}, \tag{1}$$

where PIV represents the prescription isodose volume, and TV_{PIV} is the target volume that received the PIV.GI describes the steepness of dose falloff outside the target [2], estimated as:

$$\mathrm{GI} = \frac{\mathrm{PIV}_{50\%}}{\mathrm{PIV}}, \tag{2}$$

where $PIV_{50\%}$ represents the volume enclosed by half of the PIV. Plans with similar conformity measures could be differentiated based on their GI. An ideal value of GI for a treatment plan has been proposed as <3.0 [3].

SI measures the rate of the surrounding normal tissue spared from the irradiation, and it was calculated as:

$$\mathrm{SI} = \frac{\mathrm{TV_{PIV}}}{\mathrm{PIV}}. \tag{3}$$

An ideal value of SI has been proposed as >0.9 [3]. The last dose planning index, EI ($\eta_{50\%}$), is the ratio of the energy delivered to the target volume to the total energy [4], calculated by dividing the integral dose enclosed in the target volume by the integral dose of $PIV_{50\%}$:

$$\eta_{50\%} = \frac{Integral\ dose_{TV}}{Integral\ dosePIV_{50\%}}. \tag{4}$$

EI combines the conformity, gradient, and mean target dose measurements into a single metric, ranging from 0 to 1, where 1 indicates a perfect treatment plan.

Feature Extraction

Seven shape features were extracted using delineated contours to characterize 3D model of tumors. Surface-to-volume ratio (SVR), flatness, elongation, spherical disproportion, and compactness, whose definitions are provided in Table S1, were extracted using the Radiomics extension of 3D-Slicer software. Further information about radiomic features can be accessed online [5].

SVR increases with tumor irregularity, having larger surface areas for a given volume. In computerized shape analysis, it is preferable to describe objects using metrics robust to scaling and rotation. SVR was normalized (SVR_N) by multiplying it by the average of the three principal semi-axes of the tumor model ($a, b, and\ c$; $r = (a + b + c)/3$, Figure 1), since SVR is proportional to $^1/_r$.

The sphericity degree is defined as:

$$Sphericity = \frac{TV}{V_{CS}} = \sqrt[3]{\frac{d_{SV}}{d_{CS}}}, \tag{5}$$

where V_{cs} is the volume of the smallest circumscribed sphere that would fit into the tumor, and d_{SV} and d_{CS} are the diameters of spheres that would have the same volume as the target and the circumscribing sphere, respectively [6].

Finally, VioS [7] measures the tumor shape divergence from a perfect sphere, computed as:

$$VioS = \frac{I_{AS}}{I_{IS}}, \tag{6}$$

where I_{IS} is the SVR of a sphere that would have the same volume as the tumor, and I_{AS} is the SVR of the actual shape computed as $4\pi r^2 / \frac{4}{3}\pi r^3$. The r value was estimated as the average of the three principal semi-axes (Fig. S1).

Table S1. Description of five radiomic features extracted by the tumor models.

	Description	Formulation
**Surface to volume ratio**	The ratio of the surface area (A) to the volume of the model (V). A lower value indicates a sphere-like shape.	$\frac{A}{V}$
**Compactness**	Quantifies how compact the tumor's shape is, relative to a sphere represented by a value of 1.	$36\pi \frac{V^2}{A^3}$
**Flatness**	Shows the ratio of the lengths of the largest and smallest principal axes. (1 for non-flat object/sphere and 0 for flat object/single slice of the segmented region)	$\sqrt{\frac{\lambda_{least}}{\lambda_{major}}}$
**Elongation**	The ratio of the major (λ_{major}) and minor (λ_{minor}) axes of the segmented volume. A value of 1 indicates that it is not elongated (ccircle-like and 0 represents a maximally elongated object.	$\sqrt{\frac{\lambda_{minor}}{\lambda_{major}}}$
**Spherical Disproportion**	The ratio of the surface area of the tumor to the surface area of a sphere with the same volume as the tumor. A value of 1 reflects a perfect sphere (R is the sphere's radius).	$\frac{A}{4 \times \pi \times R^2}$

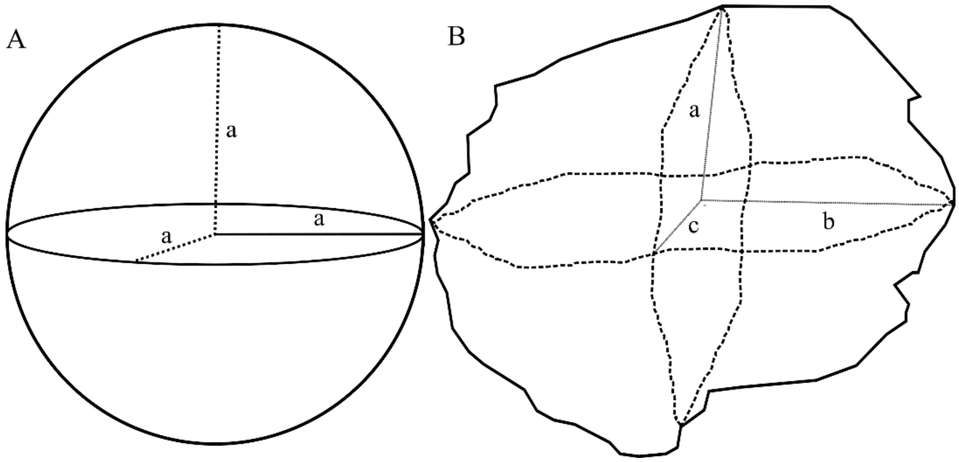

Fig. S1. A perfect sphere (a = b = c) was given in A. The brain tumors are not perfect spheres, and they have different axis lengths as depicted in B (a ≠ b ≠ c) (adapted from Saad *et al.*[7])

Table S2. Univariate linear regression analysis results using VioS. (*Abbreviations:* SI= selectivity index, PCI= Paddick's conformity index, GI= gradient index, EI= efficiency index.)

Response	Predictor	Estimate± std	*P*-value	95% CI	Adj. R^2
**SI**	Intercept	0.6649±0.020	3.05E-87	(0.626, 0.704)	0.272
	Slope	0.2485±0.028	1.21E-16	(0.194, 0.303)	
**GI**	Intercept	2.9312± 0.031	1.47E-174	(2.870,2.992)	0.242
	Slope	-0.3593±0.043	1.27E-14	(-0.445,-0.274)	
**EI**	Intercept	0.3419±0.013	1.23E-70	(0.317,0.367)	0.377
	Slope	0.1995±0.018	1.67E-23	(0.165,0.234)	
**PCI**	Intercept	0.6175±0.018	1.71E-88	(0.582, 0.653)	0.331
	Slope	0.2636±0.025	1.03E-20	(0.214, 0.314)	

Table S3. The results of univariable and multivariable analyses for examining Gardner-Robertson serviciable hearing preservation after GK radiosurgery. (*Abbreviations:* TV=treatment volume, CI= coverage index, SI= selectivity index, GI= gradient index, EI= efficiency index, VioS= volumetric index of sphericity.)

	**Univariable Analysis**		**Multivariable Analysis**	
**Covariate**	HR (95% CI)	*P*-value	HR (95% CI)	*P*-value
**Age**	1.01 (0.97, 1.04)	0.70	1.00 (0.97, 1.04)	0.94
**Sex**	0.72 (0.30, 1.77)	0.48	0.72 (0.28,1.86)	0.50
**Koos**	1.28 (0.80, 2.04)	0.30	1.36 (0.58, 3.15)	0.48
**Dynamic shaping**				
**TV**	1.08 (0.72, 1.61)	0.72	0.63 (0.26, 1.54)	0.31
**Margin dose**	1.00(0.64,1.56)	1.00	0.98 (0.61, 1.56)	0.93
**Cochlea-target distance**	1.05 (067,1.66)	0.82	1.16 (0.62, 2.17)	0.64
**CI**	1.09 (0.68, 1.73)	0.73	1.76 (0.70, 4.43)	0.23
**SI**	1.32 (0.76, 2.29)	0.32	0.53 (0.04, 7.20)	0.64
**GI**	0.82 (0.48, 1.40)	0.48	1.65 (0.40, 6.88)	0.49
**EI**	1.34 (0.81, 2.21)	0.26	4.29 (0.18, 100.28)	0.37
**Maximum cochlear dose**	1.01(0.65,1.56)	0.98	1.27 (0.62, 2.63)	0.51
**VioS**	1.36 (0.81, 2.28)	0.24	1.17 (0.58, 2.39)	0.66

Table S4. The results of univariable and multivariable analyses examining tumor growth control after GK radiosurgery. (*Abbreviations:* TV= treatment volume, CI= coverage index, SI= selectivity index, GI= gradient index, EI= efficiency index, VioS= volumetric index of sphericity.)

	**Univariable Analysis**		**Multivariable Analysis**	
**Covariate**	HR (95% CI)	*P*-value	HR (95% CI)	*P*-value
**Age**	1.00 (0.97, 1.03)	0.88	0.98 (0.95, 1.01)	0.28
**Sex**	0.63 (0.27, 1.51)	0.30	0.55 (0.23, 1.33)	0.18
**Koos**	0.84 (0.52, 1.35)	0.47	0.58 (0.26, 1.26)	0.17
**TV**	1.01 (0.67, 1.53)	0.96	0.91 (0.46, 1.82)	0.79
**Margin dose**	0.71(0.51, 1.00)	0.05	0.60 (0.41, 0.89)	0.01
**CI**	0.81 (0.58, 1.13)	0.21	1.04 (0.47, 2.31)	0.92
**SI**	1.57 (0.89, 2.79)	0.12	2.08 (0.26, 16.53)	0.49
**GI**	1.15 (0.79, 1.68)	0.46	1.13 (0.35, 3.66)	0.84
**EI**	1.32 (0.82, 2.12)	0.25	1.08 (0.06, 17.96)	0.96
**VioS**	1.16 (0.75, 1.80)	0.51	1.07 (0.57, 2.03)	0.82

References:

1 Paddick, I. A simple scoring ratio to index the conformity of radiosurgical treatment plans - Technical note. *J Neurosurg* **93**, 219-222, doi:10.3171/jns.2000.93.supplement_3.0219 (2000).

2 Paddick, I. & Lippitz, B. A simple dose gradient measurement tool to complement the conformity index. *J Neurosurg* **105**, 194-201, doi:10.3171/sup.2006.105.7.194 (2006).

3 Torrens, M. *et al.* Standardization of terminology in stereotactic radiosurgery: report from the Standardization Committee of the International Leksell Gamma Knife Society. *J Neurosurg* **121**, 2-15, doi:10.3171/2014.7.Gks141199 (2014).

4 Dimitriadis, A. & Paddick, I. A novel index for assessing treatment plan quality in stereotactic radiosurgery. *J Neurosurg* **129**, 118-124, doi:10.3171/2018.7.GKS18694 (2018).

5 Pyradiomics. *Welcome to pyradiomics documentation!*, https://pyradiomics.readthedocs.io/en/latest/# (

6 Chagas Saraiva, C. W. *et al.* Gamma Knife radiosurgery for vestibular schwannomas: evaluation of planning using the sphericity degree of the target volume. *PLoS One* **15**, e0225638, doi:10.1371/journal.pone.0225638 (2020).

7 Saad, M., Lee, I. H. & Choi, T. S. Are shape morphologies associated with survival? A potential shape-based biomarker predicting survival in lung cancer. *J Cancer Res Clin Oncol* **145**, 2937-2950, doi:10.1007/s00432-019-03048-1 (2019).
